# Improving the Cost-Effectiveness of Artificial Visual Baits for Controlling the Tsetse Fly *Glossina fuscipes fuscipes*

**DOI:** 10.1371/journal.pntd.0000474

**Published:** 2009-07-07

**Authors:** Jenny M. Lindh, Steve J. Torr, Glyn A. Vale, Mike J. Lehane

**Affiliations:** 1 Vector Group, Liverpool School of Tropical Medicine, Liverpool, United Kingdom; 2 ICIPE, Thomas Odhiambo Campus, Mbita Point, Kenya; 3 Natural Resources Institute, University of Greenwich, Chatham, Kent, United Kingdom; 4 South African Centre for Epidemiological Modelling and Analysis (SACEMA), University of Stellenbosch, Stellenbosch, South Africa; ICIPE, Kenya

## Abstract

Tsetse flies, which transmit sleeping sickness to humans and nagana to cattle, are commonly controlled by stationary artificial baits consisting of traps or insecticide-treated screens known as targets. In Kenya the use of electrocuting sampling devices showed that the numbers of *Glossina fuscipes fuscipes* (Newstead) visiting a biconical trap were nearly double those visiting a black target of 100 cm×100 cm. However, only 40% of the males and 21% of the females entered the trap, whereas 71% and 34%, respectively, alighted on the target. The greater number visiting the trap appeared to be due to its being largely blue, rather than being three-dimensional or raised above the ground. Through a series of variations of target design we show that a blue-and-black panel of cloth (0.06 m^2^) flanked by a panel (0.06 m^2^) of fine black netting, placed at ground level, would be about ten times more cost-effective than traps or large targets in control campaigns. This finding has important implications for controlling all subspecies of *G. fuscipes*, which are currently responsible for more than 90% of sleeping sickness cases.

**Author summary**

Sleeping sickness remains a serious threat to many of the poorest people in Africa. Tsetse flies transmit the trypanosome species that cause the disease. There are no vaccines or prophylactic drugs to prevent people from contracting the disease, which is dealt with after it has been contracted using drugs that are often ineffective and in addition have unpleasant and sometimes fatal side effects. Prospects for development of effective vaccines or prophylactic drugs are poor. Killing tsetse flies can prevent disease transmission either locally (e.g., a group of villages) or regionally (covering large parts of a country or region). One important means of killing tsetse flies is to use insecticide-treated cloth screens known as targets. However, a major problem is the cost and logistical difficulty of implementing such fly control programs. To overcome this obstacle, we are trying to develop more cost-effective insecticide-treated targets. Here we show that the major vector, *Glossina fuscipes fuscipes*, is attracted to very small targets (25 cm^2^) provided with the same area of flanking netting. This system is about ten times more cost-effective than the traps or large targets currently used. This finding has important implications for controlling all subspecies of *G. fuscipes*, which are currently responsible for more than 90% of sleeping sickness cases.

## Introduction

Tsetse flies (*Glossina* spp.) transmit the fatal diseases of sleeping sickness in humans and the cattle disease nagana. Tsetse flies are commonly divided into three ecologically distinct groups: savannah tsetse ( = Morsitans group), which are largely responsible for transmitting the trypanosomes that cause the cattle disease nagana; riverine tsetse ( = Palpalis group), which play a major role the transmission of *Trypanosoma brucei* spp., the causative agents of human sleeping sickness; and forest tsetse (Fusca group) which, generally speaking, do not play an important epidemiological role.

The absence of vaccines, and problems with the availability, toxicity, and resistance to drugs [1] mean that controlling the vector is a highly attractive means of tackling the diseases. One of the most important methods of tsetse control is the use of stationary artificial baits that simulate host animals and consist either of three-dimensional traps or cloth screens that are treated with insecticide and known as targets [2]. The recommended targets are black, blue, or blue/black, about 1.0–1.7 m^2^ and, for the savannah species of tsetse, they are baited with odor attractants and deployed at a density of about four per square kilometer. For most of the riverine species of tsetse, traps rather than targets are commonly used and, since no effective odor attractants are known for these flies, the required density of baits is relatively great (>10/km^2^). Hence, the cost of controlling riverine tsetse using artificial baits is at least twice that for the savannah flies [3]. Nevertheless, the use of artificial baits is favored for controlling riverine tsetse, partly because it is cheaper than methods such as the sterile insect technique and aerial spraying [3], and because it is suitable for community implementation [4]. Hence, any economies in the bait control of riverine species would be particularly welcome.

So far, attempts to improve bait control of the riverine tsetse have concentrated largely on traps, especially in the case of *Glossina fuscipes fuscipes* [5],[6],[7], which together with the other two subspecies of *G. fuscipes* are implicated in more than 90% of sleeping sickness cases [8],[9]. Moreover, with all riverine species the refinement of targets has focused mainly on color and materials [10],[11],[12], not size. The present work with *G. f. fuscipes* elucidates the relative effectiveness of traps and a wide variety of targets, with particular attention to size, and demonstrates much potential for the use of small targets in control operations.

## Materials and Methods

Studies were performed from August 2007 to December 2008 on the 0.5 km^2^ of Chamaunga Island (0°25′S, 34°13′E), Lake Victoria, Kenya. Baits consisted of a blue biconical trap [13] and targets made from cotton cloth dyed black or Phthalogen blue (reflectance spectra for the cloth are included in Figure S1). Electrocuting grids placed over fine black netting were also placed next to targets and traps where they intercepted flies in flight—the so-called flanking nets. The fine black polyester net (Quality no. 166, Swisstulle, Nottingham, UK) and the electrocuting wires of the electric net used here are effectively invisible to tsetse [14],[15]. Electrocuted flies fell into trays of soapy water below the grids. When no flanking nets were used, the catches in the trap, and those made by grids on the target cloth, indicated the numbers of flies that would be killed in field campaigns to control tsetse by traps or insecticide-treated targets. However, to understand the full potential for improving bait performance it was necessary to know also what proportion of the flies that visited the baits actually entered or alighted before departing, i.e., the efficiency of the baits. To assess this, the number of flies visiting the baits was taken as the catch in the trap, or on the target, plus the catch of a flanking net. Efficiency of the trap or target was then calculated as the number of flies at the baits themselves, as a percentage of the number visiting.

Experiments were carried out between 09.00 and 13.00 h, when *G. f. fuscipes* is most active [16],[17], using a series of Latin-squares of days×sites×treatments, with sites at least 50 m apart. Analysis of variance was performed after transforming the catches to log (n+1), the significance of differences between means being assessed by the Student-Newman-Keuls (SNK) test when more than two means were compared.

## Results

### Distinctions between traps and targets

#### Trap versus target

**Figure 1:**
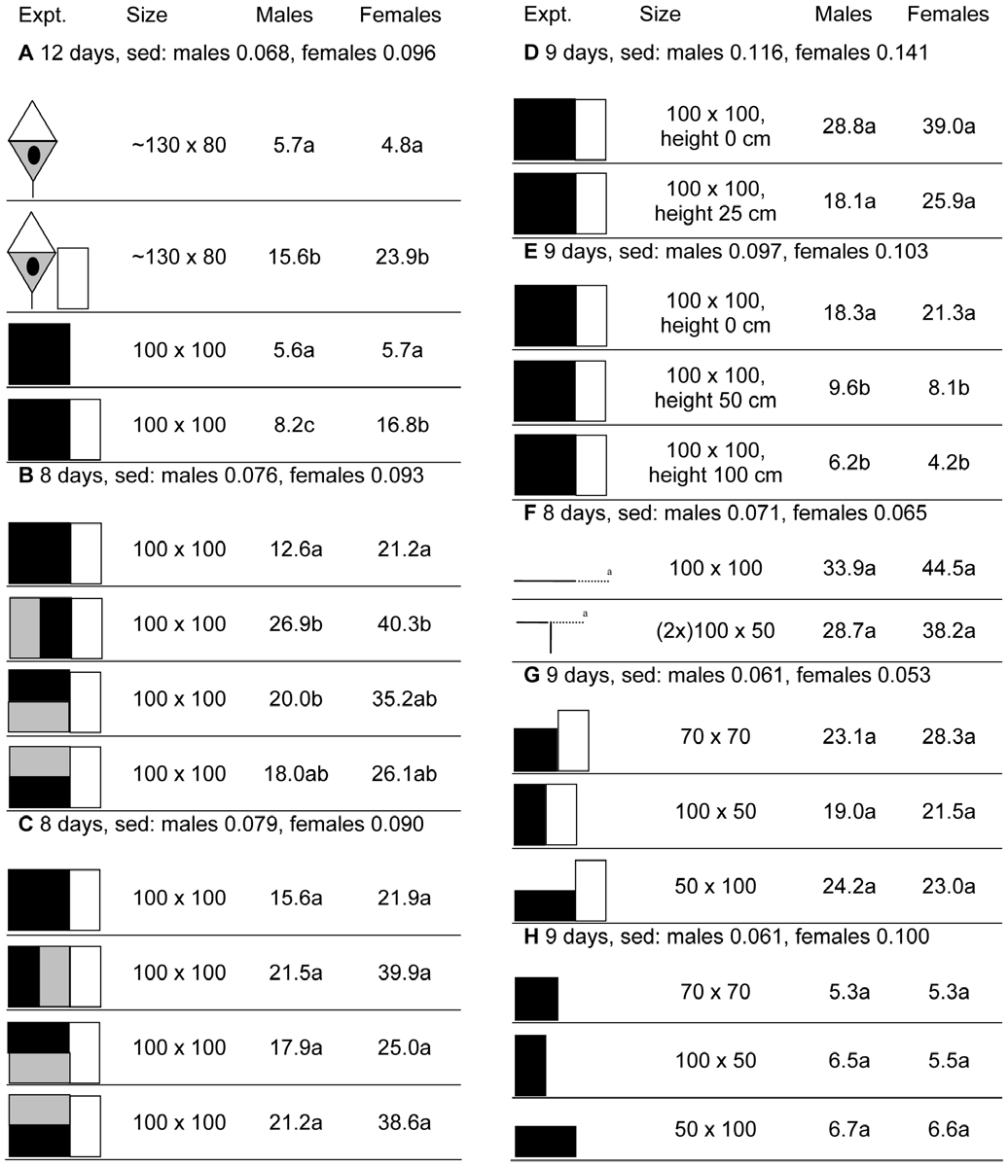
Detransformed mean catches in eight experiments investigating distinctions between traps and targets. Standard error of differences (sed) refer to transformed means, which are not shown. In each experiment, means not associated with the same letter differ at *p*<0.05. Panels: white = netting; black = black cloth; grey = blue cloth. Size (height×width) refer to the overall cloth component, height in cm refers to the height above the ground at which the target was placed. Figures are proportional in size. ^a^Plan view of cloth (solid line) and net (dotted line).

Biconical traps typify the sorts of trap used to control *G. f. fuscipes* and other riverine tsetse [5],[11],[12],[18]. A 100×100 cm black target is the common benchmark for target performance with several species [19]. These two baits were compared in the presence and absence of flanking nets of 100×50 cm (all dimensions are reported as height×width). With the nets, the total catches suggested that the trap attracted 1.9 times as many males and 1.4 times as many females as the target, although the effect was significant only with the males (Figure 1, exp. A). However, comparison between the catches with and without the net showed that the trap efficiency (as defined in Materials and Methods) was only 40% for males and 21% for females, as against efficiencies of 71% and 34% respectively for the target. So, in the absence of the nets the catches at the baits were roughly similar. Ideally the target should be at least as attractive as the trap, while also maximizing the alighting response. Hence, the next few studies assessed how the performance of targets was affected when they were modified to appear more like the trap.

#### Color

The results of experiments B and C of Figure 1 together indicate that the blue/black targets were up to twice as attractive as the all black target, although the effect was significant only when the blue and black panels were vertical and the black panel was next to the net. However, when the data were reanalyzed to compare black versus blue/black targets, the differences were significant in both experiments for females (exp B: P = 0.027, F = 5.7, standard error of differences [sed] = 0.080, detransformed means: black = 21.2, blue/black = 33.4; exp C: P = 0.035, F = 5.1, sed = 0.081 detransformed means: black = 21.9, blue/black = 33.8) and in exp B for males (exp B: P = 0.040, F = 10.7, sed = 0.066, detransformed means: black = 12.6, blue/black = 21.4; exp C: P = 0.114, F = 2.7, sed = 0.063, detransformed means black = 15.6, blue/black = 20.1). Some part of the superior attractiveness of the trap seems due to its being largely blue.

#### Height above ground

Experiments D and E of Figure 1 showed that raising the target 25 cm off the ground had little effect on target performance, but attractiveness decreased steadily and significantly at a greater height, to be reduced by 76% for males and 80% for females at 100 cm. Hence, the fact that the biconical trap was mounted about 40–50 cm off the ground (its normal positioning in trapping operations in the area) cannot explain its greater attractiveness.

#### Three dimensions

The normal two-dimensional black target, 100×100 cm, was compared with a target composed of two black panels, 100×50 cm, joining each other at a right angle. The results (exp F, Figure 1) showed no benefit of making the target three-dimensional, i.e., more like the trap. A complication in this experiment was that when the three-dimensional target was viewed from the angle that maximized its apparent width it was slightly oblong, not square like the target with which it was compared. This suggested that the effect of oblongs should be explored further.

#### Shape

To study the effect of the shape of two-dimensional targets it was convenient to reduce the target size by half, to ∼0.5 m^2^, so that the square target was 70×70 cm and the oblong had sides of 100 cm and 50 cm. The results, using the standard 100×50 cm flanking net (exp G, Figure 1), suggested that attractiveness was not affected by target shape. However, given that shape is known to affect the alighting responses of savannah tsetse [20],[21], it seemed necessary to compare also the performance of the variously shaped targets when catches were restricted to alighting flies only, i.e., no flanking nets, only grids on the targets. It appeared (exp H, Figure 1) that even when considering alighting flies only, the shape of targets was unimportant.

Taking all of the above results together, it seemed that the greater attraction to the blue biconical trap, relative to the black target, was probably primarily due to color distinction, as expected from studies with several other species of tsetse [22],[23],[24]. But more work on color distinction is required with this species before firm conclusions can be drawn. It was more intriguing that the catches from the black targets of only 0.5 m^2^ were not much lower than those from the black targets of 1 m^2^, whether the flanking nets were present or not, i.e., whether or not catches were determined by alighting responses alone. This observation was confirmed in another experiment were the catch on a flanking net (100×50 cm) next to a 0.5 m^2^ (100×50 cm) black target were 63% and 57% of those on a flanking net next to a 1 m^2^ black target for males and females respectively (exp A, Figure S2). This contrasts sharply with the data for the savannah tsetse [19],[25] that indicate that size reductions decrease performance greatly, due especially to weaker alighting responses. Thus, the following experiments explored further the effect of target size and means of enhancing the performance of small targets.

### Optimization of small targets

#### Size reduction

**Figure 2:**
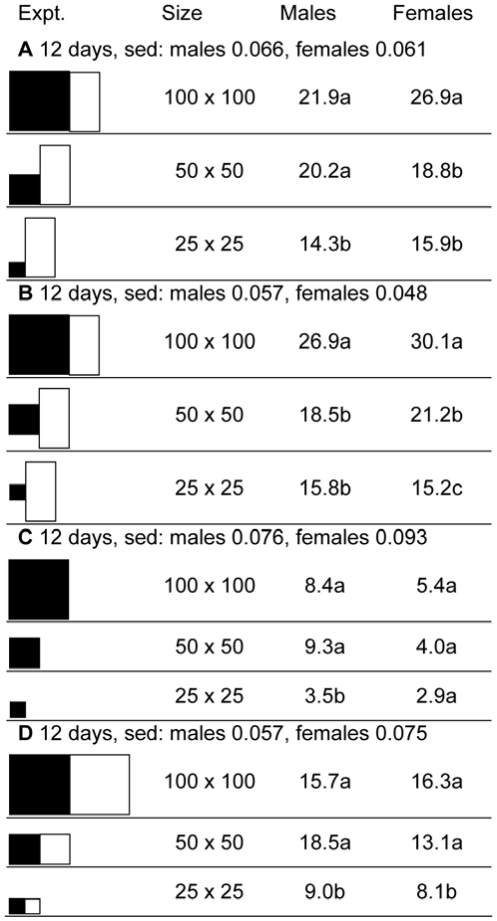
Detransformed mean catches in four experiments investigating the effect of target size. Standard error of differences (sed) refer to transformed means, which are not shown. In each experiment, means not associated with the same letter differ at *p*<0.05. Panels: white = netting; black = black cloth; grey = blue cloth. Size (height×width) refer to the overall cloth component. Figures are proportional in size.

The four experiments of Figure 2 used square black targets to assess how much target size could be reduced without a significant reduction in catch. Sometimes the smaller targets were raised off the ground, so that their centers of visual conspicuousness were at the same height as that for the large target on the ground. The salient point was that reducing the target size to 25×25 cm, i.e., to 1/16th of the area of the large target, gave catches that declined remarkably little, by a mere half on average, suggesting that the cost-effectiveness of per square centimeter of cloth would be enhanced about 8-fold by using tiny targets. Hence, further work concentrated on mostly the 1/16th-sized targets (0.0625 m^2^), although the biconical trap and/or the large (1 m^2^) black target were sometimes included to keep sight of the fact that an important criterion for any new target is its performance relative to more standard baits.

#### Shape

**Figure 3:**
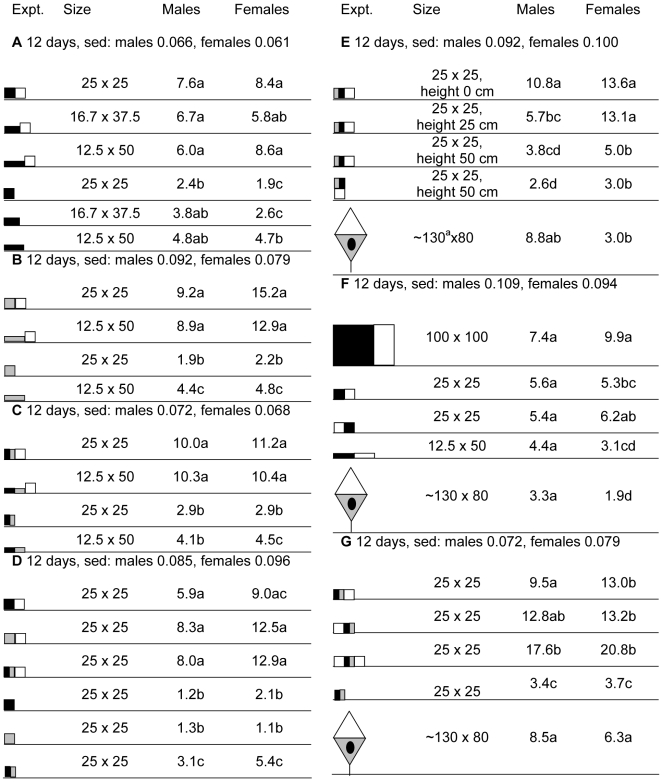
Detransformed mean catches in seven experiments aiming to optimize the design of small targets. Standard error of differences (sed) refer to transformed means, which are not shown. In each experiment, means not associated with the same letter differ at *p*<0.05. Panels: white = netting; black = black cloth; grey = blue cloth. Size (height×width) refers to the overall cloth component. Height in cm refers to height above ground at which the target was placed. Figures are proportional in size.

In experiments A–C of Figure 3 the catches from the horizontal oblong target were about double those from the square when only alighting flies were caught, i.e., when nets were absent. This effect was significant for females in all cases, but was significant for males only with the blue targets. However, when the targets were used with a flanking net, to assess the number of flies visiting the baits, there was a smaller and less consistent effect of shape, suggesting that the oblongs induced stronger alighting responses. This is confirmed by the pooled results of all three experiments (exp A–C, Figure 3), which show that the percentage of flies alighting on the squares was 29% for males and 20% for females, as against 49%–57% for males and 44%–48% for females on the two oblongs.

#### Color

In exp D of figure 3 the catches with nets present were increased by about a third when the target was all-blue or blue/black instead of all-black. These effects were not significant, but they approximate to the effects of color with large targets (exp B and C, Figure 1). However, with the small targets in the absence of nets (exp D, Figure 3), the blue/black target caught several times more males and females than either the all-black or all-blue, and the effects were significant. The implication is that the percentage of flies alighting on the small blue/black target was 43% for males and 37% for females, compared with only 21%–24% for males and 15%–23% for females on the two small monochromes.

#### Height above ground

In accord with the indications from the height study with large black targets (exp D and E, Figure 1), increasing the height of small blue/black targets to 50 cm reduced their catches significantly, by half for males and three-quarters for females (exp E, Figure 3). The more remarkable observation was that the catch at the small blue/black target on the ground compared favorably with that from the trap, showing no significant difference for males but a significant 4- to 5-fold improvement for females.

#### Further comparison with standard baits

The final two experiments (exp F and G, Figure 3) confirmed that a variety of small targets with a small net gave catches that were: (i) about half of those from the large black target, (ii) about the same as trap catches for males, and (iii) several times greater than trap catches for females. Experiment G of Figure 3 emphasizes that the net panel can be an important feature of small targets, since catches declined significantly, by about two-thirds, when the panel was removed; catches increased by about half when an extra panel of net was added, although the effect was not significant.

## Discussion

The present work shows that targets can be designed to catch several times more *G. f. fuscipes* than traps; such targets are much cheaper and simpler than traps, and easier to maintain. These observations confirm the long-standing generalization, based on studies with other tsetse species, that targets are much more cost-effective than traps [2]. Strikingly, the present work suggests that very small and therefore highly cost-efficient targets are suitable for *G. f. fuscipes*.

Comparison of the effects of target size on various species of tsetse is complicated since the available sets of data refer to targets of different shape, color, and elevation, and sometimes with electrified nets of distinctive size and arrangement [11],[12],[20],[25],[26]. Nevertheless, targets of much less than about 1 m^2^ are strongly contraindicated for the savannah tsetse, *G. pallidipes* (Austen) and *G. morsitans morsitans* (Westwood), the species for which size effects have been analyzed most [19],[25]. One problem with small targets is that relatively few savannah tsetse visit them, but a more important problem is that the probability of the flies alighting on them can be very poor, especially for females, the sex that is most important to attack in control campaigns. For example, with *G. pallidipes* and a black cylindrical target of about 0.2 m^2^, the percent alighting was only 1.2% for males and 0.5% for females, as against figures of 33.9% and 33.3%, respectively, for a target of similar color and shape but nine times the area [25]. However, in the present work, panels of only 0.0625 m^2^ attracted remarkably large numbers of *G. f. fuscipes*, and the percentage alighting on such tiny targets of the better shape and color was around 40%–55%, which is much the same as for large targets. In any case, it seems that small panels of fine, insecticide-treated net added to the side of the small cloth panels could offset the problem that some tsetse would not contact insecticide on the cloth. The same principle applies with the savannah tsetse [19], but the correspondingly larger sheets of netting needed with the large targets used for these flies are particularly prone to damage. Moreover, with the savannah species the added panels of net are hardly better than added cloth panels of about the same size, since the extra visual stimulus greatly improves the strength of the alighting response. For example, extra panels of cloth to increase the target size by eight times, from 0.25 to 2.00 m^2^, enhanced the alighting catch by about 30-fold for female *G. m. morsitans* and 100-fold for female *G. pallidipes* [19]. In contrast, the present work shows that increasing the cloth size by 16 times improved the alighting catch of female *G. f. fuscipes* (exp C, Figure 2) by a mere 86%. Viewed another way, the number of *G. m. morsitans* and *G. pallidipes* killed per cm^2^ of cloth (an important aspect of cost-effectiveness) dropped to virtually nil as the cloth size declined toward 0.1 m^2^, whereas for *G. f. fuscipes* the number increased about 10-fold.

More should be done to optimize target design for *G. f. fuscipes*, and to make fuller and more critical comparisons with other species, but it is already clear that the cost-effectiveness of target operations against *G. f. fuscipes* could be improved substantially by using small targets with a little netting adjacent. The cost of materials, insecticide, and transport would decline by about 90%, and the convenience of deploying each target would be enhanced. These improvements would more than offset the fact that twice as many targets would be needed to maintain efficacy. Moreover, with such small, inexpensive targets it might be acceptable to make them disposable and biodegradable, giving further improvements in convenience. Smaller targets, made of less-durable materials, would be less prone to theft. Furthermore, reduction in the cost and operational difficulties of bait operations is itself the key to extra economies since it improves the opportunities for community involvement, which avoids many of the substantial overheads that can burden government work [3].

Currently available evidence that target shape is important for tiny targets (exp A–C, Figure 3) but not large ones (exp G and H, Figure 1) warns against assuming no interaction between target size and other features. For example, although the performance of large targets for Morsitans group flies is not improved by allowing them to swivel in the wind [19], such movement could be important with smaller and inherently less conspicuous baits. Additionally, while odor attractants released at large targets have proved much less effective for riverine tsetse than for savannah species, it could be expected that odors might be more useful with smaller targets. For example, lizard odor doubled the numbers of *G. f. fuscipes* landing on a small tube (as well as a larger target) [27],[28]. Presumably, the distinctively strong response of *G. f. fuscipes* to tiny targets relates to this species feeding often on lizards [16] rather than on the large, active, and relatively scarce herbivores that dominate the diet of savannah tsetse [29]. Hence, other aspects of the host-finding behavior of *G. f. fuscipes* can also be expected to be adapted for the discovery of small, abundant, and poorly mobile hosts, perhaps involving a relatively close quartering of the habitat, which has implications for the appropriate spacing and siting of baits.

In conclusion, present indications for the performance of relatively tiny targets suggest the need for new thinking, re-exploration, and wider studies, not only with *G. f. fuscipes* but also other riverine tsetse fly species.

## Supporting Information

Figure S1 Reflectance spectra for the target cloth utilized in the study (Mbita blue and Mbita black respectively). A spectrum for a Phthalogen blue cloth (#40, Phthalogen blue) utilized in previous studies on visual responses of other *Glossina* species is included for comparison [10].
(0.09 MB TIF)

Figure S2 Detransformed mean catches of flies caught on flanking net only. Standard error of differences (sed) refer to transformed means, which are not shown. Means not associated with the same letter differ at P<0.05. Panels: white = netting; black = black cloth. Size (height×width) refers to the overall cloth component. Figures are proportional in size.
(0.07 MB TIF)
