# Supplementary figures and images for: Improving the Cost-Effectiveness of Artificial Visual Baits for Controlling the Tsetse Fly Glossina fuscipes fuscipes
Source: PLoS Negl Trop Dis. 2009 Jul 7;3(7):e474. doi: 10.1371/journal.pntd.0000474 (PMC2699553; doi:10.1371/journal.pntd.0000474)

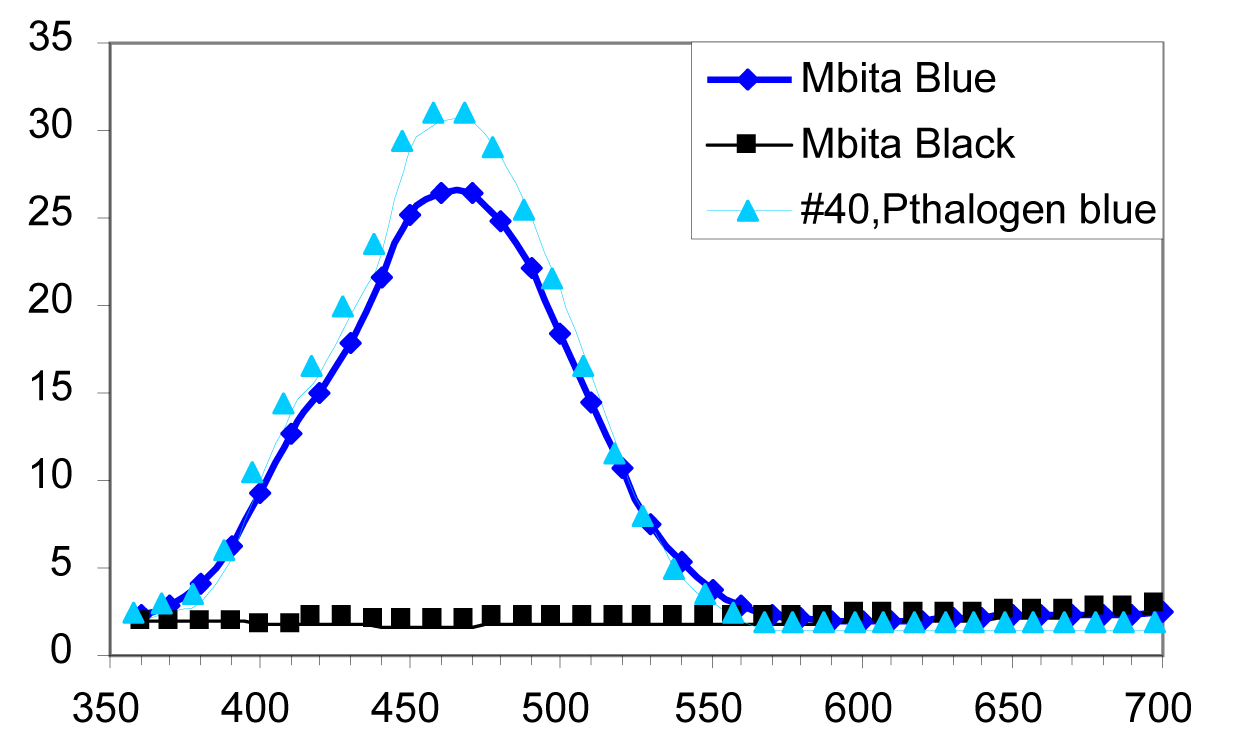

Supplement: Figure S1 — Reflectance spectra for the target cloth utilized in the study (Mbita blue and Mbita black respectively). A spectrum for a Phthalogen blue cloth (#40, Phthalogen blue) utilized in previous studies on visual responses of other Glossina species is included for comparison [10]. (0.09 MB TIF) [file pntd.0000474.s001.tif]

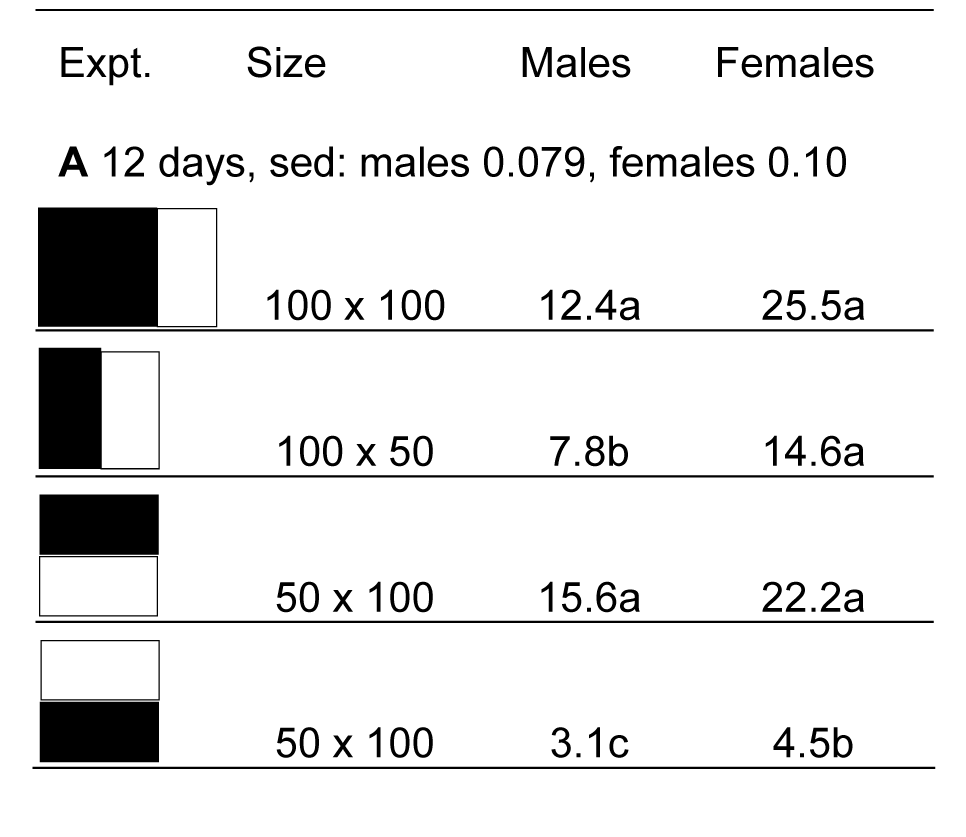

Supplement: Figure S2 — Detransformed mean catches of flies caught on flanking net only. Standard error of differences (sed) refer to transformed means, which are not shown. Means not associated with the same letter differ at P<0.05. Panels: white = netting; black = black cloth. Size (height×width) refers to the overall cloth component. Figures are proportional in size. (0.07 MB TIF) [file pntd.0000474.s002.tif]
